# Short- and Long-Term Survival Prediction Using Different Prognostic Scores in Cardiovascular Surgeries

**DOI:** 10.3390/jcm15072760

**Published:** 2026-04-06

**Authors:** Alexandros C. Liatsos, Styliani Ioakeimidou, Mairi Panagidi, Andreas S. Papazoglou, Dimitrios V. Moysidis, Athanasios Samaras, Fani Tsolaki, Georgios I. Tagarakis

**Affiliations:** 1 Medical School, Aristotle University of Thessaloniki, 54124 Thessaloniki, Greece; alexliatso@yahoo.gr (A.C.L.);; 2 Department of Internal Medicine, University Hospital of Zurich, 8091 Zurich, Switzerland; 3 Department of Cardiothoracic Surgery, AHEPA University Hospital of Thessaloniki, 54636 Thessaloniki, Greece; pan.mary@hotmail.com (M.P.);; 4 Department of Cardiology, Athens Naval Hospital, 15561 Athens, Greece

**Keywords:** EuroSCORE II, SOFA score, APACHE II score, Karnofsky score, risk stratification, postoperative mortality

## Abstract

**Background**: Early identification of patients at risk for adverse outcomes after cardiac surgery remains a major clinical challenge. While preoperative risk scores are widely used, the prognostic value of early postoperative ICU severity scores and functional performance measures has not been fully clarified. **Methods**: This prospective observational study included 195 patients undergoing cardiac surgery between 2018 and 2024. Predictive performance of EuroSCORE II, the SOFA score, the APACHE II score, Karnofsky performance status, handgrip strength, and phase angle was assessed for postoperative complications and mortality. Receiver operating characteristic (ROC) curves with 95% confidence intervals were calculated, and pairwise comparisons between ROC curves were performed. Major postoperative complications were analyzed using a composite endpoint including stroke, prolonged intubation, sepsis, and reoperation, excluding systemic inflammatory response syndrome (SIRS). **Results**: Major postoperative complications occurred in 46 patients (23.6%). For prediction of major postoperative complications, SOFA demonstrated the highest discrimination (AUC = 0.881, 95% CI 0.819–0.928), followed by APACHE II (AUC = 0.826, 95% CI 0.753–0.888) and EuroSCORE II (AUC = 0.695, 95% CI 0.602–0.785). In-hospital mortality occurred in 19 patients (9.7%). SOFA showed the strongest predictive performance (AUC = 0.915, 95% CI 0.851–0.968), followed by APACHE II (AUC = 0.869, 95% CI 0.781–0.939) and EuroSCORE II (AUC = 0.742, 95% CI 0.595–0.870). During follow-up, 54 patients (27.7%) died. Predictive performance was comparable between SOFA (AUC = 0.710, 95% CI 0.618–0.793), APACHE II (AUC = 0.695, 95% CI 0.606–0.782), and EuroSCORE II (AUC = 0.680, 95% CI 0.599–0.757). **Conclusions**: Early postoperative ICU severity scores, particularly SOFA and APACHE II, demonstrated strong predictive ability for major postoperative complications and in-hospital mortality following cardiac surgery and outperformed preoperative risk scores.

## 1. Introduction

Cardiovascular surgeries, including procedures such as coronary artery bypass grafting (CABG) and valve replacements, are critical interventions for patients with severe cardiovascular diseases. The optimization of clinical decision-making as well as patient outcomes is highly driven by and dependent on a correct prediction of the postoperative survival of each patient [1]. This goal is mostly accomplished through a variety of developed and validated prognostic scores, such as EuroSCORE II, the Karnofsky score, the SOFA score and the APACHE II score, which estimate the risk of mortality and complications by using multiple clinical variables, thus leading to a comprehensive risk assessment [2]. Surgical planning in conjunction with resource allocation and postoperative care strategies are qualitatively enhanced by such prognostic models in a proportional way.

The aforementioned scores have been well established in the medical literature in terms of their prognostic value in cardiovascular surgeries [3]. The European System for Cardiac Operative Risk Evaluation (EuroSCORE) II, a widely used in Europe scoring system, has been associated with reliable predictive capabilities regarding mortality and postoperative complications in cardiac surgery patients [4]. Although it has been a robust predictive tool for many years, the need for regional adjustments has been stressed with a view toward elevating its prognostic accuracy across diverse populations [5]. Recent studies have suggested that incorporating specific demographic and clinical factors unique to different regions could significantly improve the ability of EuroSCORE II to accurately reflect patient outcomes, thereby fostering more personalized approaches to cardiac care [6].

A typically oncologic score, the Karnofsky score, has been also studied in the context of cardiovascular surgeries, in attempts to be used for evaluating patients’ functional status and predicting postoperative outcomes [7]. Lower survival rates and more complications have been correlated with lower Karnofsky scores, underlying its relevance beyond its traditional use [8].

Moreover, the Sequential Organ Failure Assessment (SOFA) score and the Acute Physiology and Chronic Health Evaluation II (APACHE II) score have also found utility in cardiovascular surgical patients, although they were originally developed for critically ill patients in intensive care. The SOFA score, which considers organ dysfunction and septic complications, has shown strong predictive value for postoperative morbidity and mortality in patients undergoing complex cardiac procedures [9]. In the same axis, the APACHE II score has been validated as a significant predictor of adverse outcomes in cardiac surgery patients, by assessing physiological derangements, chronic health status, and age [10].

Handgrip Strength is a simple, non-invasive measure of skeletal muscle function and overall physical capacity, while Phase Angle is derived from bioelectrical impedance analysis and reflects cellular health, membrane integrity, and nutritional status. Both parameters are increasingly used as adjuncts to traditional risk scores for perioperative assessment [11]. In cardiothoracic surgery patients, low Handgrip Strength and a low Phase Angle have been independently associated with increased risk of postoperative complications, prolonged ICU and hospital length of stay, and higher short- and long-term mortality [12].

In this context, it is clear that cardiovascular surgery patients could be significantly benefited by the proper handling of these prognostic scores. A simultaneous study of all these scores in terms of their utility as survival and complications’ predictors for operated cardiovascular surgeries is still needed in the current literature. Attempting to bridge this gap, the current study aims to investigate and compare the prognostic value of different scores under the scope of survival prediction in a real-world cohort of patients undergoing cardiac surgery.

## 2. Materials and Methods

### 2.1. Study Design and Study Population

The current trial involved adult patients who were hospitalized in the cardiothoracic surgery ward of a tertiary hospital and had undergone a cardiovascular operation for coronary artery disease, namely CABG, cardiac valve disease, namely aortic valve replacement (AVR), or both. The current study source population consists of 195 patients hospitalized between 21 February 2018 and 15 February 2019, in fact in the course of approximately one year. Patients suffering from other cardiac conditions or patients that had undergone types of surgeries other than the above-mentioned, as well as patients who presented factors that would obstruct their follow up, such as missing data, fulfilled the exclusion criteria of the study and were consequently considered ineligible for its purpose.

### 2.2. Study Data and Definitions

Prospective data from this patient dataset were gathered for the needs of the current analysis. Baseline demographic characteristics, such as age and gender, clinical profiles, medical history, laboratory data, discharge diagnoses, and medications, formed the electronic database of the study. All patients were examined for existing comorbidities, among them most often arising diabetes mellitus (DM), chronic obstructive pulmonary disease (COPD) and chronic kidney disease (CKD). Moreover, the length of stay in an intensive care unit (ICU) and the duration of mechanical ventilation (in days) were both documented.

Furthermore, more specialized tests were performed in patients to form a reliable patient profile and through this evaluate the progression of their medical status. These tests measured the bioelectrical impedance of patients using Phase Angle, which is the tan value of the ratio of reactance versus electric resistance [13], as well as their maximum voluntary muscle strength through Handgrip Strength, with which patients’ functionality to perform daily activities was reflected [14]. The collection of all these data was performed by trained independent physicians who interviewed and examined patients while they were hospitalized and had access to their discharge notes and their full hospital record.

We calculated 4 types of prognostic scores, EuroSCORE II, the Karnofsky score, the SOFA score and the APACHE II score, on the first postoperative day (POD 1). EuroSCORE II was calculated preoperatively using standard clinical and operative variables according to the original scoring system. For the calculation of EuroSCORE II, the calculator provided in the official EuroSCORE website was used [15]. On the contrary, all other scores were calculated through the reliable MD + CALC application [16]. Postoperative APACHE II and SOFA scores were calculated within the first 24 h after ICU admission, using the worst physiological values recorded during that period, in accordance with established scoring methodology. Functional and frailty measures were assessed once patients were clinically stable and able to cooperate, also within postoperative day 1. From a clinical perspective, early identification of patients at increased risk for adverse outcomes following cardiac surgery is crucial for optimizing postoperative management. The present findings suggest that routine calculation of ICU severity scores such as SOFA and APACHE II on postoperative day 1 may provide valuable prognostic information beyond traditional preoperative risk assessment.

### 2.3. Study Outcomes

**Figure 1:**
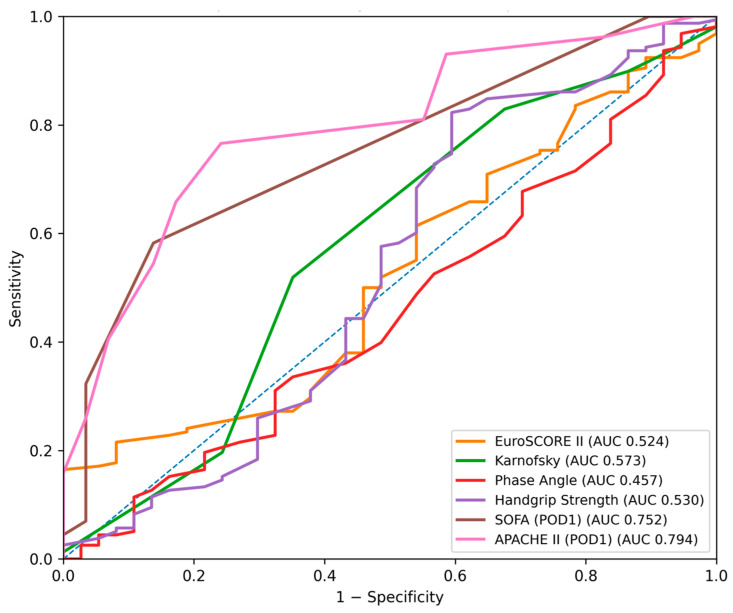
Receiver operating characteristic (ROC) curves for the prediction of major postoperative complications (including systemic inflammatory response syndrome (SIRS)) using EuroSCORE II, the SOFA score (postoperative day 1), the APACHE II score, the Karnofsky score, Phase Angle and Handgrip Strength. The diagonal dashed line represents no discriminative ability.

**Figure 2:**
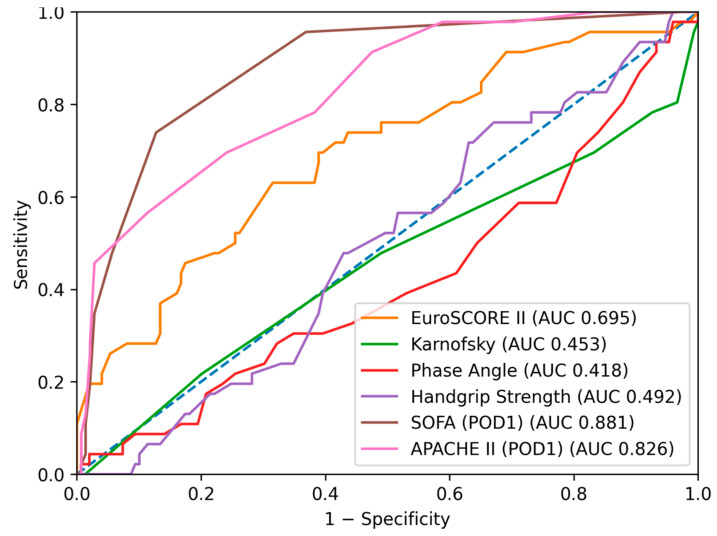
Receiver operating characteristic (ROC) curves for the prediction of major postoperative complications (excluding systemic inflammatory response syndrome (SIRS)) using EuroSCORE II, SOFA score (postoperative day 1), APACHE II score, Karnofsky score, Phase Angle and Handgrip Strength. The diagonal dashed line represents no discriminative ability.

The primary outcome in the current study was the assessment of all-cause mortality and its correlation with the predictive capability of these 4 prognostic scores and 2 frailty indicators. Mortality assessment was performed in two separate timeframes, in-hospital and out-hospital, namely, follow-up and mortality. Secondary outcomes of the study constituted any confirmed postoperative in-hospital adverse events. The latter included systematic inflammatory response syndrome (SIRS), sepsis, stroke, prolonged intubation and reoperation (Figure 1 and Figure 2). As far as out-hospital mortality is concerned, patients were followed annually from the index date of hospital discharge via use of the insurance number of each patient, given after the written consent. In this way, we ascertained all deaths by the Greek electronic insurance system. The follow-up period ended in February 2024 after the last search for each patient.

Postoperative outcomes were defined according to established consensus criteria. Prolonged intubation was defined as the need for mechanical ventilation for more than 48 h after surgery, in accordance with previously published guidelines on ventilatory support and weaning [17]. Systemic inflammatory response syndrome (SIRS) was defined based on the criteria established by the American College of Chest Physicians and the Society of Critical Care Medicine consensus conference, requiring the presence of at least two of the following: body temperature > 38 °C or <36 °C, heart rate > 90 beats/min, respiratory rate > 20 breaths/min or PaCO_2_ < 32 mmHg, and white blood cell count > 12,000/mm^3^, <4000/mm^3^, or >10% immature forms [18].

Sepsis was defined according to the Third International Consensus Definitions for Sepsis and Septic Shock (Sepsis-3) as life-threatening organ dysfunction caused by a dysregulated host response to infection, operationalized as an increase in the Sequential Organ Failure Assessment (SOFA) score of ≥2 points in the presence of suspected or confirmed infection [19].

Stroke was defined according to the World Health Organization criteria as a rapidly developing clinical sign of focal or global disturbance of cerebral function lasting more than 24 h or leading to death, with no apparent cause other than vascular origin [20]. Reoperation was defined as an unplanned return to the operating room during the index hospitalization for management of a postoperative complication.

According to the central study outcomes, patients’ data were grouped into 3 different binomial categories, directly associated with the study’s aim. In the first, the criterion was whether the patients demonstrated any adverse in-hospital event following the surgery or not. The second category revolved around in-hospital mortality, regardless of the cause of death, whereas in the third category patients were clustered based on follow-up mortality.

### 2.4. Statistical Analysis

Continuous variables are presented as median with interquartile range (IQR) and categorical variables as counts with percentages. Continuous variables were compared using the Mann–Whitney U test, while categorical variables were compared using the chi-square test or Fisher’s exact test as appropriate. Discriminative ability of the examined scores was assessed using receiver operating characteristic (ROC) curve analysis with calculation of the area under the curve (AUC) and corresponding 95% confidence intervals. Pairwise comparisons between ROC curves were performed to evaluate differences in predictive performance. Parsimonious multivariable logistic regression models were constructed to evaluate associations between risk scores and mortality outcomes after adjustment for age, sex, surgical procedure, and chronic kidney disease. The number of variables was limited according to the number of outcome events to reduce the risk of model overfitting. Statistical significance was defined by a *p*-value of less than 0.05 using two-sided tests. R version 4.4.0 (R Foundation for Statistical Computing, Vienna, Austria) and JAMOVI version 2.5.4 statistical software were used to perform all analyses.

## 3. Results

A total of 195 patients undergoing cardiovascular surgery at AHEPA University Hospital, Thessaloniki, were included in the analysis. The median age of the cohort was 67 years (IQR: 15), and 75.3% were male. Surgical procedures comprised coronary artery bypass grafting (CABG) in 86 patients (44.1%), aortic valve surgery (AVR) in 88 patients (45.3%), and combined CABG with valve surgery in 19 patients (10.0%).

### 3.1. In-Hospital Adverse Events

Overall, 170 patients (87.1%) experienced at least one adverse in-hospital postoperative event (Table 1). These events included systemic inflammatory response syndrome (SIRS), sepsis, prolonged mechanical ventilation, and reoperation. SIRS and extended intubation (82.9% and 23.5%, respectively) were the most frequent adverse events recorded. When SIRS was excluded from the composite endpoint, major postoperative complications occurred in 46 patients (23.6%), including prolonged intubation, stroke, sepsis, or reoperation.

Patients with adverse events had a significantly longer median ICU stay compared to those without events (*p* = 0.028), as well as a longer median hospital stay (*p* = 0.015). The SOFA score (*p* = 0.013) and the APACHE II score (*p* < 0.001) were significantly higher in the adverse event group, while EuroSCORE II, the Karnofsky score, Phase Angle, and Handgrip Strength did not differ significantly (Table 1).

**Figure 3:**
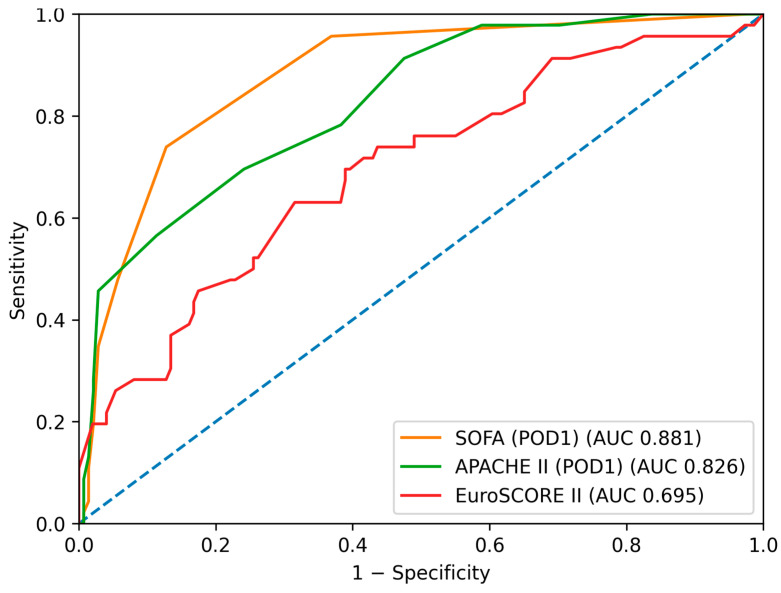
Receiver operating characteristic (ROC) curves for the prediction of major postoperative complications (excluding systemic inflammatory response syndrome (SIRS)) using EuroSCORE II, the SOFA score (postoperative day 1), and the APACHE II score. The diagonal dashed line represents no discriminative ability.

**Figure 4:**
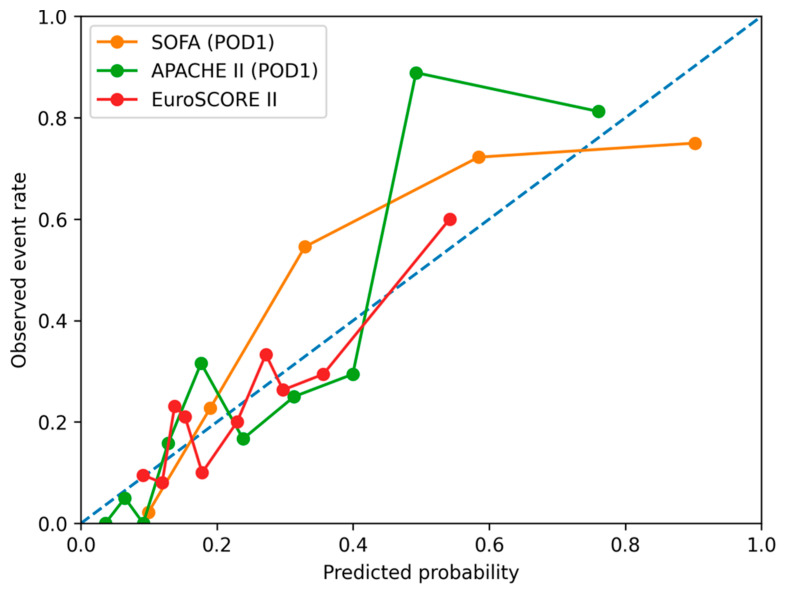
Calibration plots for the prediction of major postoperative complications (excluding systemic inflammatory response syndrome (SIRS)) using EuroSCORE II, the SOFA score (postoperative day 1), and the APACHE II score. The diagonal dashed line represents no discriminative ability.

ROC curve analysis for prediction of any adverse in-hospital event (Figure 1, Figure 2, Figure 3 and Figure 4) demonstrated the highest discriminative ability for SOFA (AUC = 0.881, 95% CI: 0.819–0.928) and APACHE II (AUC = 0.826, 95% CI: 0.753–0.888). Moderate discrimination was observed for EuroSCORE II (AUC = 0.695, 95% CI: 0.602–0.785), while the Karnofsky score (AUC = 0.453, 95% CI: 0.354–0.563), Phase Angle (AUC = 0.418, 95% CI: 0.322–0.517), and Handgrip Strength (AUC = 0.492, 95% CI: 0.400–0.582) demonstrated limited predictive performance (Table 2A). Pairwise Delong comparisons confirmed the statistically significant superiority of SOFA and APACHE II compared with the other indices (Table 3A).

### 3.2. In-Hospital Mortality

Nineteen patients (9.7%) died before hospital discharge (Table 4). Compared to survivors, non-survivors had markedly prolonged ICU stays (*p* < 0.001) and ventilation times (*p* < 0.001). Postoperative scores were significantly higher among non-survivors: EuroSCORE II (*p* < 0.001), SOFA (*p* < 0.001), and APACHE II (*p* < 0.001). The Karnofsky score (*p* = 0.040) and Handgrip Strength (*p* = 0.022) were lower in patients who died in-hospital, while the Phase Angle was not significantly different.

**Figure 5:**
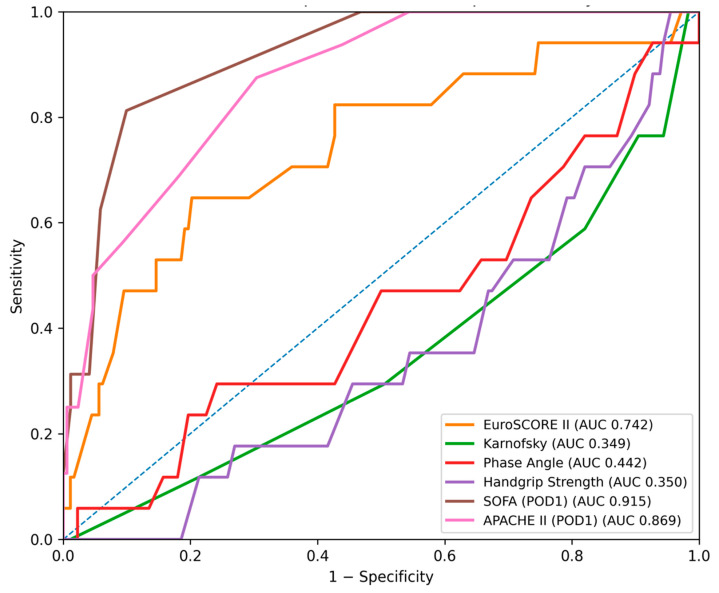
Receiver operating characteristic (ROC) curves for the prediction of in-hospital mortality using EuroSCORE II, the SOFA score (postoperative day 1), the APACHE II score, the Karnofsky score, Phase Angle and Handgrip Strength. The diagonal dashed line represents no discriminative ability.

**Figure 6:**
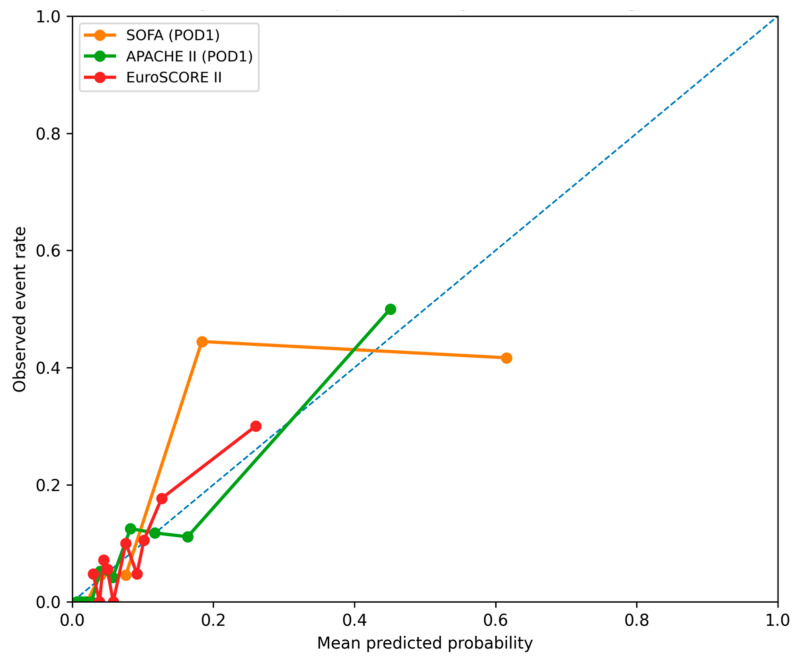
Calibration plots for the prediction of in-hospital mortality using EuroSCORE II, the SOFA score (postoperative day 1), and the APACHE II score. The diagonal dashed line represents no discriminative ability.

In the ROC analysis (Figure 5 and Figure 6), the SOFA score demonstrated the strongest predictive performance for in-hospital mortality (AUC = 0.915, 95% CI: 0.851–0.968), followed by APACHE II (AUC = 0.869, 95% CI: 0.781–0.939) and EuroSCORE II (AUC = 0.742, 95% CI: 0.595–0.870). Functional performance measures showed substantially lower discriminative ability, including the Karnofsky score (AUC = 0.349, 95% CI: 0.214–0.498), Handgrip Strength (AUC = 0.350, 95% CI: 0.218–0.486), and Phase Angle (AUC = 0.442, 95% CI: 0.290–0.599). Pairwise comparisons confirmed that SOFA performed significantly better than EuroSCORE II, whereas differences between SOFA and APACHE II were not statistically significant.

Parsimonious multivariable logistic regression analyses demonstrated that the postoperative SOFA score (aOR 2.61, 95% CI 1.79–3.80, *p* < 0.001), the APACHE II score (aOR 1.82, 95% CI 1.43–2.32, *p* < 0.001), and EuroSCORE II (aOR 1.65, 95% CI 1.19–2.27, *p* = 0.002) were independently associated with in-hospital mortality.

In the multivariable models (Table 5), SOFA (POD1), APACHE II, and EuroSCORE II remained independently associated with in-hospital mortality. Among these models, SOFA demonstrated the strongest discrimination (adjusted AUC 0.971, 95% CI 0.937–0.992), followed by APACHE II (0.883, 95% CI 0.763–0.976) and EuroSCORE II (0.738, 95% CI 0.573–0.889).

### 3.3. Follow-Up Long-Term Mortality

No loss to follow-up was observed in the study. Fifty-four patients (27.7%) died during a median 6-year follow-up (Table 6). Compared with survivors, these patients more frequently had COPD (*p* = 0.023) and CKD (*p* = 0.001). They also had longer ICU stays (*p* = 0.004) and ventilation times (*p* = 0.013). Measures of physical and functional status were significantly lower in patients who died during follow-up: Phase Angle (*p* < 0.001), Handgrip Strength (*p* = 0.020), and the Karnofsky score (*p* = 0.021).

**Figure 7:**
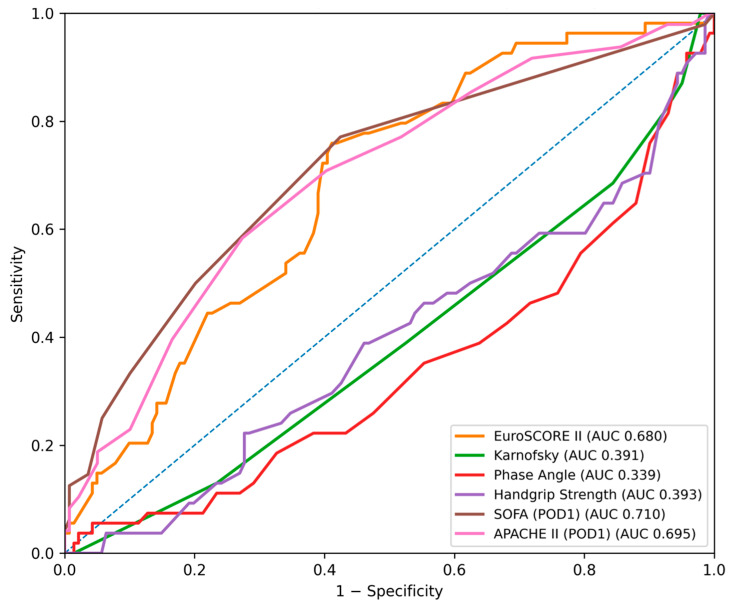
Receiver operating characteristic (ROC) curves for the prediction of follow-up mortality using EuroSCORE II, the SOFA score (postoperative day 1), the APACHE II score, the Karnofsky score, Phase Angle and Handgrip Strength. The diagonal dashed line represents no discriminative ability.

**Figure 8:**
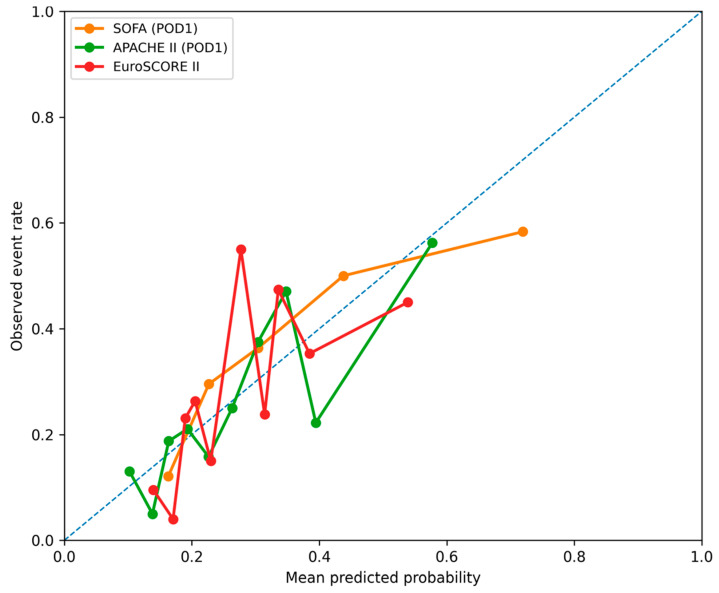
Calibration plots for the prediction of follow-up mortality using EuroSCORE II, the SOFA score (postoperative day 1), and the APACHE II score. The diagonal dashed line represents no discriminative ability.

Prognostic scores were significantly higher in the follow-up mortality group: EuroSCORE II (*p* < 0.001), SOFA (*p* < 0.001), and APACHE II (*p* < 0.001). ROC analysis (Figure 7 and Figure 8) identified SOFA (AUC = 0.710, 95% CI: 0.637–0.780), APACHE II (AUC = 0.695, 95% CI: 0.617–0.763), and EuroSCORE II (AUC = 0.680, 95% CI: 0.607–0.755) as the strongest predictors, with lower discrimination for Karnofsky, Handgrip Strength, and Phase Angle (Table 2C). Delong comparisons confirmed the superiority of SOFA, APACHE II, and EuroSCORE II over functional and physiological frailty measures (Table 3C).

For long-term mortality, multivariable models demonstrated that EuroSCORE II (aOR 1.40, 95% CI 1.08–1.82, *p* = 0.011) and the SOFA score (aOR 1.50, 95% CI 1.21–1.87, *p* < 0.001) remained independent predictors. The adjusted AUCs (Table 7) were 0.736 (95% CI 0.654–0.823) for SOFA, 0.734 (95% CI 0.650–0.815) for APACHE II, and 0.714 (95% CI 0.622–0.797) for EuroSCORE II.

### 3.4. Subgroup Analyses

Subgroup analysis by age demonstrated a higher predictive ability of EuroSCORE II and APACHE II in patients aged ≥70 years for follow-up mortality (AUC = 0.724, 95% CI: 0.618–0.750 and 0.705, 95% CI: 0.693–0.734, respectively) compared with younger patients (AUC = 0.612, 95% CI: 0.576–0.638 and 0.643, 95% CI: 0.613–0.689, respectively). The SOFA score performance remained relatively stable across age groups. The Karnofsky score demonstrated reduced predictive performance in older patients, with no meaningful improvement in AUC.

When stratified by surgical procedure, SOFA and APACHE II were more accurate in predicting in-hospital events and mortality in CABG patients (AUC = 0.769, 95% CI: 0.698–0.801, and 0.721, 95% CI: 0.701–0.745, respectively) than in valve surgery patients (AUC = 0.689, 95% CI: 0.642–0.723, and 0.678, 95% CI: 0.651–0.707, respectively). Conversely, EuroSCORE II and the Karnofsky score showed better predictive performance in valve surgery patients for follow-up mortality (AUC = 0.703, 95% CI: 0.669–0.738, and 0.672, 95% CI: 0.641–0.704, respectively).

Handgrip Strength and Phase Angle demonstrated limited predictive performance across all subgroups, with no meaningful differences according to age or surgical type.

## 4. Discussion

In this real-world observational study, we evaluated the prognostic utility of various risk scores and frailty indicators in predicting the risk of postoperative outcomes in a predominantly elderly cardiac surgery cohort including 75.3% males and a CABG/SAVR ratio of ~1:1. Postoperative complications were common, with 87.1% of patients experiencing at least one in-hospital adverse event. The most frequently observed adverse events were SIRS, sepsis, prolonged mechanical ventilation, and the need for reoperation. Pairwise comparisons showed better performance of APACHE II and SOFA over the other indices for postoperative complications, although the overall discriminative ability was moderate.

In-hospital mortality occurred in 9.7% of patients. ROC analysis demonstrated that acute physiological scores, particularly SOFA and APACHE II, showed the highest discriminative performance for predicting in-hospital mortality, while functional measures such as the Karnofsky score and Handgrip Strength showed lower predictive ability. During a median 6-year follow-up, 27.7% of patients died. In contrast to in-hospital mortality, long-term mortality was also associated with traditional prognostic scores; SOFA, APACHE II, and EuroSCORE II demonstrated the highest though modest predictive accuracy. Subgroup analyses revealed that the predictive accuracy of EuroSCORE II and APACHE II for long-term mortality was greater among patients aged ≥70 years, whereas SOFA remained relatively consistent across age groups. In terms of surgical type, SOFA and APACHE II were more effective in predicting outcomes among CABG patients, while EuroSCORE II and the Karnofsky score were more predictive in valve surgery patients [21,22]. Our findings highlight the complementary strengths of acute physiological and chronic functional assessments in predicting short- and long-term outcomes, rather than clear superiority of a single scoring system.

Acute physiological scores, particularly SOFA and APACHE II, demonstrated the highest discriminative ability for predicting in-hospital mortality in our cohort, outperforming traditional surgical risk scores such as EuroSCORE II and functional measures including the Karnofsky score and Handgrip Strength. These findings are consistent with previous research demonstrating significantly higher SOFA and APACHE II scores among patients with poor short-term prognostic course [23]. In the same axis, regarding SOFA and APACHE II, meta-analyses and large validation studies in cardiac surgery report AUCs for in-hospital mortality prediction between 0.75 and 0.85, with most falling in the 0.75–0.80 range [24,25,26]. Other approaches achieved even higher AUCs for in-hospital mortality up to 0.92 in large TAVR cohorts and 0.887 in pediatric cardiac surgery [25,26].

In a previous study by Porizka F et al., the Karnofsky performance status has also been proposed as a relevant predictor of both short- and long-term mortality, especially in valve surgery patients with baseline functional decline, with lower scores associated with increased 30-day and two-year mortality [27,28,29]. Similarly, prior multicenter findings linking reduced Handgrip Strength to increased 1-year mortality highlight the potential value of functional assessments in perioperative risk stratification [25,26].

Overall, the aforementioned scores assess acute physiological instability and organ dysfunction, thereby making them particularly relevant in the immediate postoperative period, where rapid clinical changes may influence outcomes. In our cohort, SOFA and APACHE II demonstrated the best overall performance for predicting postoperative complications, while EuroSCORE II provided additional information regarding baseline surgical risk. Overall, these scores focus on physiological derangements and preoperative risk factors, which makes them useful, though not definitive, tools in identifying patients at higher risk of poor outcomes, particularly in elderly populations [30].

Long-term mortality occurred in 27.7% of patients and was significantly associated with chronic conditions such as COPD and CKD, prolonged ICU stays, and reduced functional measures including Phase Angle, Handgrip Strength, and Karnofsky scores. Higher EuroSCORE II, SOFA, and APACHE II scores were also associated with increased long-term mortality, with SOFA achieving an AUC of 0.709 (95% CI: 0.618–0.793), APACHE II 0.690 (95% CI: 0.606–0.782), and EuroSCORE II 0.681 (95% CI: 0.599–0.757), indicating moderate discriminative performance. Functional measures showed relatively weaker discrimination for long-term outcomes. Comparing these findings to the literature, the reasonable but imperfect performance of risk scores such as SOFA, APACHE II, and EuroSCORE II for long-term mortality prediction aligns with multiple studies in cardiac surgery and critical care populations [29,31,32]. EuroSCORE II has demonstrated consistent predictive utility for morbidity and mortality, often outperforming or complementing APACHE II and SOFA scores, especially when calculated preoperatively and in postcardiac surgery ICU settings. These scores capture acute physiological derangements and organ dysfunction, which remain relevant to long-term survival likely through their reflection of baseline patient resilience and severity of illness during critical illness episodes.

Conclusively, the APACHE and SOFA scores performed in our cohort comparably to the most widely used risk scores in cardiothoracic patients, including the Global Registry for Acute Coronary Events (GRACE) risk score system, the age, creatinine and ejection fraction (ACEF) risk score system and its modified AGEF version, the SYNTAX II score, and the Canada Acute Coronary Syndrome (C-ACS) risk assessment system, confirming their reliability for risk stratification in this population [33,34,35], while acknowledging their limitations. The existing body of evidence also suggests that EuroSCORE II remains one of the better-performing models for cardiac surgery risk stratification [36,37,38,39].

Based on our findings, specific recommendations can be made regarding the clinical application of the evaluated scoring systems. For short-term prognostic assessment, particularly for predicting in-hospital mortality, ICU severity scores reflecting acute physiological derangement, such as SOFA and APACHE II, appear most suitable. For predicting overall postoperative complications, these acute physiologic scores also demonstrated superior performance and may be preferred for early postoperative risk stratification and intensive monitoring. In contrast, for long-term prognostic assessment, traditional risk models such as EuroSCORE II, SOFA, and APACHE II showed the highest predictive accuracy, particularly among patients aged ≥70 years and those with a significant comorbidity burden. Therefore, an integrated approach combining functional assessments with established physiologic and surgical risk scores may provide the most comprehensive risk stratification strategy in cardiac surgery populations.

### 4.1. Limitations

This study has several limitations that should be acknowledged. First, the relatively small sample size may have limited the statistical power to detect more subtle associations between clinical variables and patient outcomes. Second, we performed unadjusted regression analyses, which do not account for potential confounding factors that may have influenced the observed associations. Nonetheless, given the multicomponent nature of most of the assessed scores, we expect that residual confounding would be less pronounced. Moreover, our primary objective was to evaluate the predictive capacity of these scores rather than to establish causal relationships. Therefore, the results should be interpreted cautiously with respect to causality. Finally, although the Simplified Acute Physiology Score (SAPS) II has been reported to outperform SOFA and APACHE II in predicting longer-term outcomes, such as 1-year mortality [40], our study did not evaluate this score. The study did not include a systematic preoperative baseline assessment of all prognostic indices, precluding evaluation of perioperative score dynamics. Furthermore, although all measurements were performed within the first 24 h after ICU admission, variability within this time window may have influenced score precision. Another limitation of the present study is the relatively small number of in-hospital deaths (n = 19), which may limit the statistical power for robust discrimination analyses and pairwise ROC comparisons. Consequently, the estimated AUC values should be interpreted with caution, as discrimination estimates may be unstable when the number of outcome events is limited.

### 4.2. Future Directions

Recent studies highlight the potential of biomarker-based studies to enhance the prognostic accuracy [41], emphasizing the importance of patient selection and model calibration. Future research may focus on new tools/risk models that combine acute and chronic parameters. A serial risk score assessment at multiple time-points could provide additional prognostic insights [42]. Furthermore, while the sequential use of risk scores as temporal input features for AI-based models represents a promising avenue for future research, such approaches require prospective validation before clinical application. Similarly, omics-based integration into clinical risk models may offer incremental benefits, but current evidence remains insufficient to support routine implementation.

Omics are recognized as a tool to improve serial risk assessment in cardiac surgery and cardiovascular diseases. A possible integration into clinical risk models could offer several potential advantages. Studies demonstrate that the addition of biomarkers or omics data (e.g., troponin, natriuretic peptide, and gene expression scores) to established tools like EuroSCORE II or SOFA augments prognostic accuracy for short- and long-term outcomes. Machine learning applied to big data (multi-omics) can identify and weigh key molecular predictors, outperforming conventional models in predicting adverse events and facilitating individualized risk pathways. By including dynamic changes in omics biomarkers, clinicians can detect molecular shifts that precede clinical deterioration or complications.

## 5. Conclusions

Our findings support a synergistic approach to existing risk stratification scores since most of them did not achieve optimal predictive performance for both short- and long-term mortality risk. In our real-world study, acute physiological scores such as SOFA and APACHE II emerged as best suited for predicting immediate postoperative outcomes, while preoperative functional scores like EuroSCORE II and Karnofsky provided valuable insights into long-term survival.

Selecting risk assessment tools according to patient age, surgical context, and the outcomes of interest may enhance prognostic accuracy and support clinical decision-making. Nevertheless, the generally modest predictive performance of currently available scores likely reflects their lack of development or calibration for postoperative mortality risk in older adults undergoing cardiac surgery. This underscores the importance of moving toward a personalized era of risk assessment, in which the choice and adaptation of scoring systems are individualized for each patient. Incorporating dynamic longitudinal measures and biological markers may further strengthen predictive precision and better align these tools with the principles of personalized medicine.

## Figures and Tables

**Table 1:** Baseline characteristics of the study population stratified by occurrence of any postoperative complication.

Variable	Patients with Any Adverse Post-Surgical In-Hospital Event (n = 170)	Patients Without Any Adverse Post-Surgical In-Hospital Event (n = 25)	*p*-Value
Male sex, n (%)	128 (75.3)	20 (80.0)	0.62
Age, years, median (IQR)	67.0 (60–75)	64.5 (59–70)	0.11
CABG, n (%)	75 (44.1)	15 (60.0)	0.18
Valve surgery, n (%)	77 (45.3)	9 (36.0)	0.42
CABG + valve surgery, n (%)	18 (10.0)	1 (4.0)	0.33
Diabetes mellitus, n (%)	75 (44.1)	13 (52.0)	0.47
COPD, n (%)	53 (31.2)	7 (28.0)	0.78
CKD, n (%)	42 (24.7)	9 (36.0)	0.24
ICU stay, days, median (IQR)	2.0 (1–3)	1.0 (1–2)	0.028
Ventilation days, median (IQR)	1.0 (1–1)	1.0 (1–1)	0.14
Hospital stay, days, median (IQR)	9.0 (8–11)	7.5 (7–9)	0.015
Phase angle, degrees, median (IQR)	5.4 (4.9–6.1)	5.8 (5.2–6.4)	0.19
Handgrip strength, kg, median (IQR)	29.0 (23–35)	30.5 (25–37)	0.56
EuroSCORE II, median (IQR)	2.4 (1.5–3.8)	1.7 (1.1–2.9)	0.09
Karnofsky score, median (IQR)	80 (70–90)	85 (75–90)	0.21
SOFA score (POD1), median (IQR)	6 (5–7)	6 (5–6)	0.013
APACHE II score, median (IQR)	11 (9–14)	8.5 (7–10)	<0.001

**Table 2:** Predictive ability of different prognostic scores. (**A**). Any adverse in-hospital event; (**B**). In-hospital death; (**C**). Follow-up death.

Score	AUC (Unadjusted Analysis)	95% CIs (Lower–Upper)
(**A**)		
EuroSCORE II	0.695	0.602–0.785
Karnofsky	0.453	0.354–0.563
Phase Angle	0.418	0.322–0.517
Handgrip Strength	0.492	0.400–0.582
SOFA (POD1)	0.881	0.819–0.928
APACHE II (POD1)	0.826	0.753–0.888
(**B**)		
EuroSCORE II	0.742	0.595–0.870
Karnofsky	0.349	0.214–0.498
Phase Angle	0.442	0.290–0.599
Handgrip Strength	0.350	0.218–0.486
SOFA (POD1)	0.915	0.851–0.968
APACHE II (POD1)	0.869	0.781–0.939
(**C**)		
EuroSCORE II	0.680	0.599–0.757
Karnofsky	0.391	0.300–0.484
Phase Angle	0.339	0.256–0.421
Handgrip Strength	0.393	0.305–0.486
SOFA (POD1)	0.710	0.618–0.793
APACHE II (POD1)	0.695	0.606–0.782

**Table 3:** Delong ROC Test. (**A**). Any Adverse In-Hospital Event; (**B**). In-hospital death; (**C**). Follow-up death.

Pairwise Comparison (*p*-Value)	EuroSCORE II	SOFA Score	APACHE II Score	Karnofsky Score	Phase Angle	Handgrip Strength
(**A**)						
EUROSCORE II	-	<0.001	0.021	0.142	0.009	0.167
SOFA score	<0.001	-	0.030	<0.001	<0.001	<0.001
APACHE II score	0.021	0.030	-	<0.001	<0.001	<0.001
Karnofsky score	0.142	<0.001	<0.001	-	0.264	0.931
Phase Angle	0.009	<0.001	<0.001	0.264	-	0.228
Handgrip Strength	0.167	<0.001	<0.001	0.931	0.228	-
(**B**)						
EUROSCORE II	-	0.017	0.063	<0.001	<0.001	<0.001
SOFA score	0.017	-	0.151	<0.001	<0.001	<0.001
APACHE II score	0.063	0.193	-	<0.001	<0.001	<0.001
Karnofsky score	<0.001	<0.001	<0.001	-	0.195	0.970
Phase Angle	<0.001	<0.001	<0.001	0.195	-	0.208
Handgrip Strength	<0.001	<0.001	<0.001	0.970	0.208	-
(**C**)						
EUROSCORE II	-	0.374	0.611	<0.001	<0.001	<0.001
SOFA score	0.374	-	0.635	<0.001	<0.001	<0.001
APACHE II score	0.611	0.635	-	<0.001	<0.001	<0.001
Karnofsky score	<0.001	<0.001	<0.001	-	0.369	0.957
Phase Angle	<0.001	<0.001	<0.001	0.369	-	0.398
Handgrip Strength	<0.001	<0.001	<0.001	0.957	0.398	-

**Table 4:** Baseline characteristics of the study population stratified by in-hospital mortality.

Variable	Patients with In-Hospital Death (n = 19)	Patients Without In-Hospital Death (n = 168)	*p*-Value
Male sex, n (%)	15 (78.9)	128 (76.2)	0.80
Age, years, median (IQR)	72 (67–78)	67 (59–74)	0.22
COPD, n (%)	9 (47.4)	45 (26.8)	0.07
CKD, n (%)	6 (31.6)	39 (23.2)	0.42
ICU stay, days, median (IQR)	9 (6–14)	1 (1–2)	<0.001
Ventilation days, median (IQR)	8.5 (5–13)	1 (1–1)	<0.001
Phase angle, median (IQR)	5.0 (4.6–5.4)	5.4 (4.9–6.1)	0.18
Handgrip strength, kg	24 (19–29)	29 (23–35)	0.022
EuroSCORE II	3.9 (2.6–5.2)	2.3 (1.4–3.5)	<0.001
Karnofsky score	70 (60–80)	80 (70–90)	0.040
SOFA score (POD1)	10 (8–12)	6 (5–7)	<0.001
APACHE II score	16.5 (14–20)	11 (9–14)	<0.001

**Table 5:** In-hospital mortality.

Score	Adjusted OR for Score	95% CI	*p*-Value	Adjusted AUC	95% CI
EuroSCORE II	1.48	1.06–2.07	0.023	0.738	0.573–0.889
SOFA (POD1)	3.29	1.97–5.50	<0.001	0.971	0.937–0.992
APACHE II (POD1)	1.67	1.32–2.12	<0.001	0.883	0.763–0.976

**Table 6:** Baseline characteristics of the study population stratified by follow-up mortality.

Variable	Patients with Follow-Up Death (n = 54)	Patients Without Follow-Up Death (n = 136)	*p*-Value
Male sex	41 (75.9)	102 (75.0)	0.89
Age, years	70 (64–77)	67.5 (59–73)	0.07
COPD	23 (42.6)	35 (25.7)	0.023
CKD	23 (42.6)	27 (19.9)	0.001
ICU stay, days	2 (1–4)	1 (1–2)	0.004
Ventilation days	1 (1–3)	1 (1–1)	0.013
Phase angle	4.8 (4.3–5.3)	5.4 (4.9–6.0)	<0.001
Handgrip strength	25.5 (21–31)	29 (24–35)	0.020
EuroSCORE II	3.4 (2.3–4.6)	2.3 (1.4–3.2)	<0.001
Karnofsky score	70 (60–80)	80 (70–90)	0.021
SOFA score	7.5 (6–9)	6 (5–7)	<0.001
APACHE II score	13 (11–16)	11 (9–14)	<0.001

**Table 7:** Follow-up mortality.

Score	Adjusted OR for Score	95% CI	*p*-Value	Adjusted AUC	95% CI
EuroSCORE II	1.35	1.05–1.74	0.018	0.714	0.622–0.797
SOFA (POD1)	1.42	1.15–1.74	0.001	0.736	0.654–0.823
APACHE II (POD1)	1.23	1.08–1.40	0.001	0.734	0.650–0.815

## Data Availability

All study data are available from the corresponding study author upon reasonable request.
